# DWMamba: a structure-aware adaptive state space network for image quality improvement

**DOI:** 10.3389/fnbot.2025.1676787

**Published:** 2025-10-02

**Authors:** Wenjun Fu, Xiaobin Wang, Chuncai Yang, Liang Zhang, Lin Feng, Zhixiong Huang

**Affiliations:** ^1^Beijing China Coal Mine Engineering Co., Ltd., Beijing, China; ^2^Gansu Coal First Engineering Co. Ltd., Baiyin, Gansu, China; ^3^Wanyi First Mine, China Energy Baotou Energy Co. Ltd., Baotou, Inner Mongolia, China; ^4^School of Information and Communication Engineering, Dalian Minzu University, Dalian, Liaoning, China

**Keywords:** image quality improvement, multi-scenario enhancement, vision mamba, state space model, structural cue

## Abstract

Overcoming visual degradation in challenging imaging scenarios is essential for accurate scene understanding. Although deep learning methods have integrated various perceptual capabilities and achieved remarkable progress, their high computational cost limits practical deployment under resource-constrained conditions. Moreover, when confronted with diverse degradation types, existing methods often fail to effectively model the inconsistent attenuation across color channels and spatial regions. To tackle these challenges, we propose **DWMamba**, a degradation-aware and weight-efficient Mamba network for image quality enhancement. Specifically, DWMamba introduces an Adaptive State Space Module (ASSM) that employs a dual-stream channel monitoring mechanism and a soft fusion strategy to capture global dependencies. With linear computational complexity, ASSM strengthens the models ability to address non-uniform degradations. In addition, by leveraging explicit edge priors and region partitioning as guidance, we design a Structure-guided Residual Fusion (SGRF) module to selectively fuse shallow and deep features, thereby restoring degraded details and enhancing low-light textures. Extensive experiments demonstrate that the proposed network delivers superior qualitative and quantitative performance, with strong generalization to diverse extreme lighting conditions. The code is available at https://github.com/WindySprint/DWMamba.

## 1 Introduction

**Figure 1:**
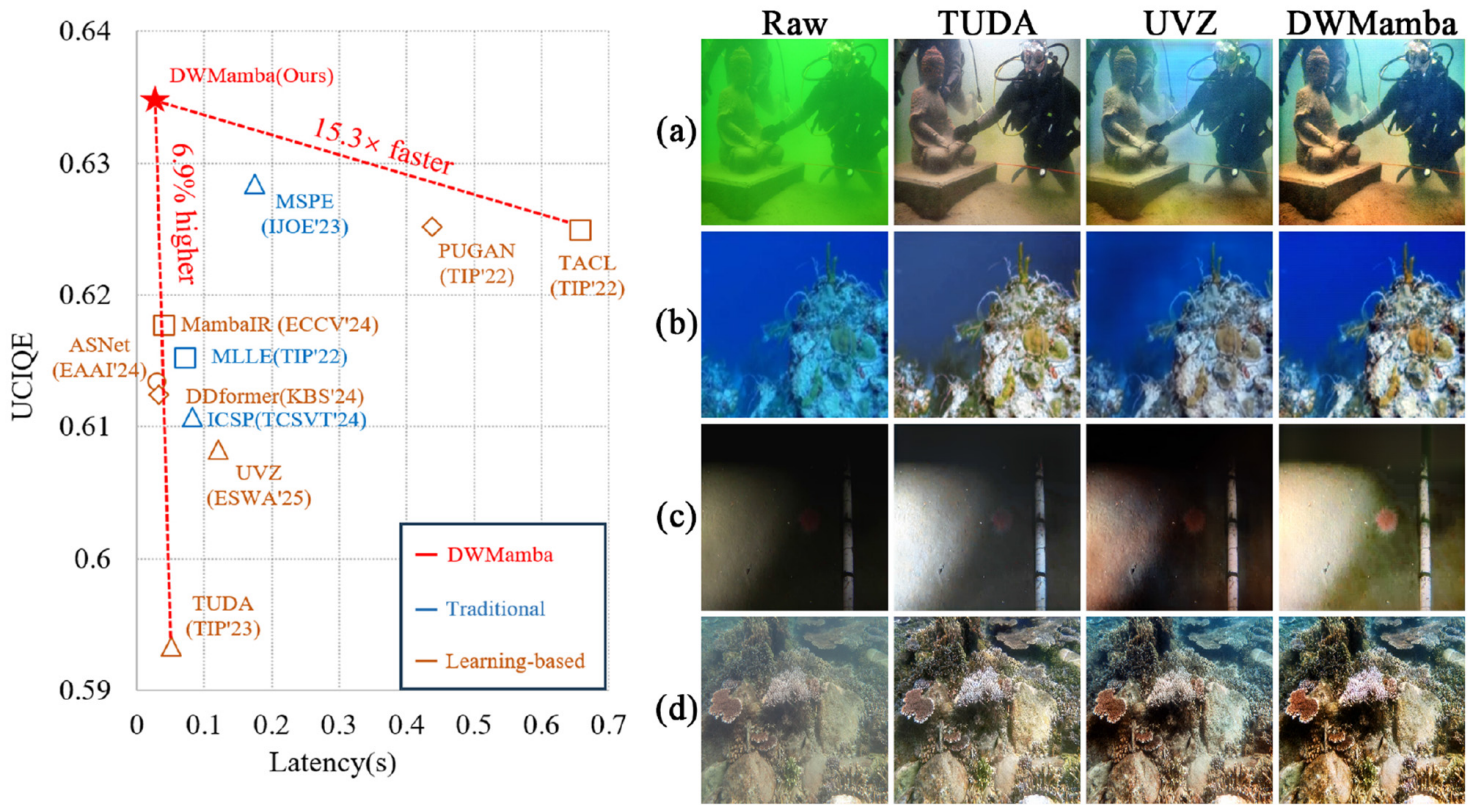
Metrics and generalizability comparison of advanced methods. The left side shows UCIQE and single-image latency. The right side shows underwater images including color deviation, low-light, overexposure, and sandy scenarios from top to bottom, along with enhanced results from different methods.

Images captured in real-world environments are often affected by various degradation factors, such as reduced visibility, blurred textures, and color distortion (Zhuang et al., 2022), which substantially hinder subsequent visual perception tasks. Developing a robust and efficient image enhancement method is therefore crucial, with two key requirements: (1) real-time performance for deployment on resource-constrained devices (Zhang et al., 2024c), and (2) strong adaptability to diverse types of degradation. Among these challenging scenarios, underwater environments represent a particularly complex case. Due to light absorption and scattering, underwater images frequently suffer from uneven brightness, amplified noise, and severe color deviation. Designing a robust enhancement method for underwater scenes is not only vital for underwater computer vision applications but also serves as a meaningful benchmark for general image enhancement. Figure 1 presents a comparison of parameters and enhancement results for several state-of-the-art methods. While some methods achieve satisfactory underwater visibility enhancement, they often fail when confronted with other severe degradations (e.g., uneven brightness or strong visual interference). In contrast, DWMamba delivers both high efficiency and robust enhancement across a wide range of challenging scenarios.

Traditional methods (Berman et al., 2020; Zhou et al., 2023c; Kang et al., 2023) aim to improve image quality by reversing the degradation process or adjusting pixel values. However, as they rely on pre-defined priors or handcrafted region partitioning, their effectiveness is often limited under variations in lighting, turbidity, and shooting conditions (Zhou et al., 2024b). In recent years, deep learning methods have received considerable attention owing to advances in computing hardware and model optimization techniques (Liu et al., 2021; Jin et al., 2022; Liu M. et al., 2024; Fan et al., 2025; Huang H. et al., 2025). Convolutional neural network (CNN)-based approaches (Fu et al., 2022; Jiang N. et al., 2022) have demonstrated strong performance in local feature extraction and achieved remarkable visibility enhancement. Nevertheless, limited by the convolutional kernel size, CNNs struggle to capture long-range dependencies, making it difficult to adequately address locally degraded regions (e.g., overexposed areas or low-light regions) in uneven degradation scenarios. To overcome this issue, Transformer-based methods (Peng et al., 2023; Khan et al., 2024) leverage global receptive fields and dynamic weights, enabling more comprehensive color correction and detail enhancement. However, their computational complexity grows quadratically with image resolution, which severely restricts their practicality in resource-constrained environments. Although some approaches attempt to improve efficiency by partitioning input regions (Chen et al., 2021; Huang et al., 2022), these strategies inevitably reduce the receptive field and impair high-level semantic understanding.

Mamba (Gu and Dao, 2023), a recently proposed selectively structured state space model, demonstrates strong capability in modeling long-range dependencies and efficiently processing long natural language sequences due to its linear computational complexity. However, when applied to 2D image sequences, Mambas limited receptive field strategy fails to capture relationships between unscanned regions. To better adapt to the visual domain, VMamba (Yue et al., 2024) introduces a four-way scanning mechanism to establish a more comprehensive receptive field. Inspired by this property, we extend VMamba to the task of image quality improvement (IQI), aiming to achieve efficient and robust visual enhancement. In practice, directly applying VMamba suffers from several limitations: (1) The absence of explicit structural cues prevents the model from adequately focusing on degradation details, particularly in low-light conditions, which limits the enhancement of degraded textures. (2) Degraded images often exhibit significant inter-channel differences, yet the model lacks a mechanism to monitor global channel dependencies during channel mapping, leading to insufficient correction of color deviations. (3) During four-way scanning, uneven regional degradation causes feature inconsistencies across different directions, and directly aggregating these results reduces the models attention to critical features. Therefore, how to further strengthen the model's perception and compensation ability for uneven degradation while maintaining efficient computation is an issue of concern.

In this work, we propose a degradation-aware and weight-efficient Mamba network for image quality improvement, called DWMamba. The framework primarily adopts an adaptive state space module (ASSM) to effectively capture global feature dependencies. Compared to VMamba, our method introduces a lightweight channel monitoring mechanism before the scanning mechanism, and implements a fusion learning strategy based on directional properties, which proves to be beneficial for the completeness of global dependencies. Furthermore, we designed a structure-guided residual fusion module (SGRF), which employs explicit edge priors and region partitioning as cues to guide the targeted fusion of shallow and deep features. Extensive comparative experiments demonstrate that DWMamba achieves competitive enhancement results with ideal computational resource. Moreover, we extended the model to different lighting environments (e.g., low-light, overexposure, haze, and sandy scenarios) without additional training, the impressive visual improvements further validate the robustness of DWMamba. The main contributions of this paper are summarized as follows:

- We present DWMamba, a novel Mamba-based network that integrates an adaptive state space module and a structure-guided residual fusion module (SGRF), offering new insights for IQI model design.
- We design an Adaptive State Space Module (ASSM) that incorporates a dual-stream channel monitoring mechanism and a soft fusion strategy, enabling finer-grained dependency modeling for accurate degradation restoration.
- We conduct extensive experiments showing that DWMamba achieves state-of-the-art performance on multiple benchmark datasets with lower computational cost, and demonstrates strong generalization across diverse visual degradation scenarios.

## 2 Related work

### 2.1 Traditional methods

Traditional methods can be broadly categorized into two types: restoration-based and enhancement-based methods. Restoration-based methods restore the input image by simulating degradation imaging and reversing this process. Dark channel prior (DCP) (He et al., 2011) provided insights into the image pixel distribution, the works (Peng et al., 2018; Liang et al., 2021) successfully extended DCP to various degraded scenarios. To address differences in channel attenuation, the works (Berman et al., 2020; Zhou et al., 2024d) introduces multiple priors to obtain higher quality images. Enhancement-based methods improve image quality through rational region partitioning and pixel adjustment. By focusing on color loss and pixel balancing, the works (Ancuti et al., 2019; Zhou et al., 2023a) effectively addressed the problems of color distortion and backscattering. Utilizing various image decomposition and fusion strategies, the works (Kang et al., 2023; An et al., 2024; Mishra et al., 2024) achieved multi-level enhancement of degraded images.

However, due to reliance on pre-set prior knowledge or single statistical feature, traditional methods may fail to generate the desired results in out-of-range scenarios.

### 2.2 Learning-based methods

With powerful feature learning capabilities and reasonable data matching, deep learning methods demonstrate good visual enhancement effects. The work (Liu M. et al., 2024) integrates two transformers that focused on multi-scale and global features, enhancing the network's response to attenuated color channels and degraded spatial regions. Introducing the color compensation mechanism, the works (Liu C. et al., 2024; Wang Y. et al., 2024) provide reasonable enhancement for the severely degraded channels, resulting in more natural images. The works (Li et al., 2021; Jiang Z. et al., 2022; Zhou et al., 2024c; Zhang et al., 2024a) adopt the multi-branch architecture to enrich feature representation, enabling the network to flexibly address the complex degradation characteristics. The works (Zhang et al., 2024d; Zhao et al., 2024) introduce the frequency domain to refine the image details, further extending the representation abilities of models.

To reduce the requirement of training data, unsupervised techniques or cross-domain knowledge transfer are applied to IQI tasks. The work (Jiang Q. et al., 2022) employs transfer learning for translating underwater images to the air domain, and then uses shared dehazing weights to further enhance the images. The work (Nathan et al., 2025) combines the in-air natural outdoor dataset with the imaging model to train the diffusion model, and obtains restored images through multiple rounds of denoising. By using target detection or evaluation metrics as training rewards, the works (Liu et al., 2022; Wang H. et al., 2024) generate enhanced images conducive to related tasks. The work (Fu et al., 2022) reconstructs raw image by estimating the relevant elements in the imaging process while utilizing the homology constraint to supervise image quality.

Deep learning methods have made significant progress in IQI tasks, but high parameters limit their practicality in resource-limited environments. More critically, the generalization of these methods is greatly challenged by variations in lighting conditions.

### 2.3 State space models

State Space Models (SSMs), as a classical sequence modeling structure in control theory, recently received extensive attention in the field of deep learning. Structured SSM (S4) (Gu et al., 2021) is a pioneering work in deep state space modeling, significantly improved modeling efficiency by improving the structure of the state matrix. Building on S4, Mamba (Gu and Dao, 2023) introduces a selectivity mechanism to break the constraints of constant transition, allowing SSM to pass or forget correlations between elements along the sequence. To introduce Mamba's properties into the visual domain, VMamba (Yue et al., 2024) proposes a cross-scan module to apply the four-way scanning mechanism, which fully expands the restricted receptive field. Inspired by the outstanding performance of VMamba, various low-level vision tasks [e.g., super-resolution Guo et al., 2025; Shi et al., 2025, low-light enhancement Bai et al., 2024; Liu et al., 2025, image dehazing Zhou et al., 2024a, and image denoising Liu Y. et al., 2024] quickly saw a boom in applications. Meanwhile, the work (Chen and Ge, 2024) first introduces the VMamba in the underwater image enhancement field and achieves high accuracy with a small number of parameters. In addition, the works (Guan et al., 2024; Dong et al., 2024) introduce the channel interaction mechanisms to capture the dependencies, while the work (Zhang et al., 2024b) introduces a physical imaging model to constrain the enhancement process, these methods maintain the linear remote modeling capability and effectively cope with wavelength-dependent channel attenuation.

However, existing VMamba-based IQI methods lack explicit cues to guide the targeted enhancement of local degradation details. Additionally, scanning features exhibit significant directional characteristics, and directly adding them results in insufficient attention to important features.

## 3 Methodology

### 3.1 Overall architecture

**Figure 2:**
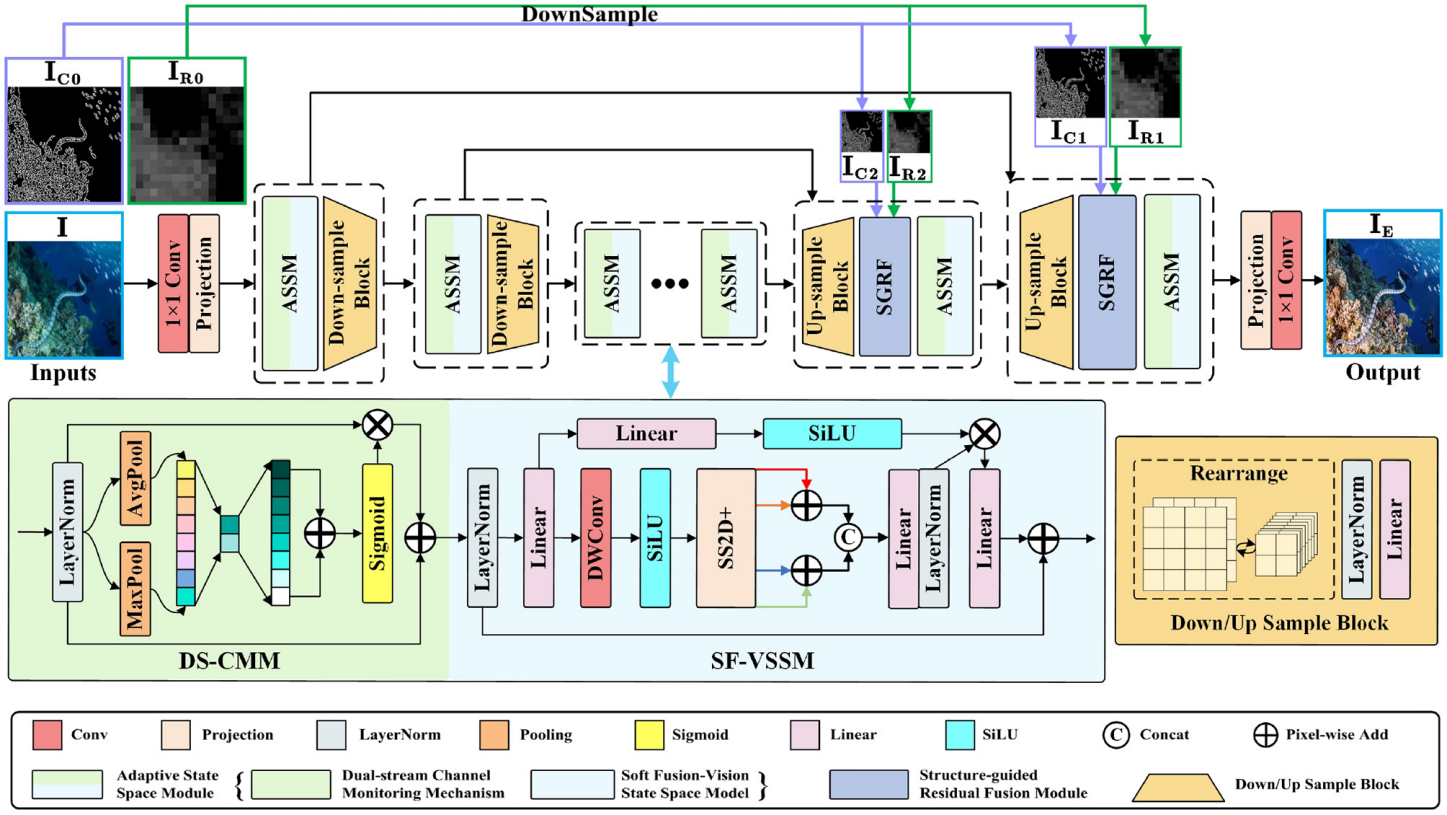
Network architecture of our proposed DWMamba. The **left** side below figure shows the detailed structure of the Adaptive State Space Module (ASSM), including the Dual-stream Channel Monitoring Module (DS-CMM) and the Soft Fusion Visual State Space Module (SF-VSSM); the **right** side shows the sample block. The details of the Structure-guided Residual Fusion Module (SGRF) are shown in Figure 5.

As shown in Figure 2, DWMamba adopts a multi-scale UNet architecture and contains a three-stage feature extraction process. For the degraded image ***I***∈ℝ^*H*×*W*×3^, we first utilize 11 convolution and channel projection to obtain the feature embedding $E_{0}\in {\mathbb{R}}^{H\times W\times C_{0}}$, where *H* and *W* represent the height and width, respectively, and *C*_0_ represents the initial number of expanded channels. Subsequently, the features are passed through two layers of symmetric encoder-decoder, each containing ASSM and corresponding sampling operation. In the lightweight network, we configure the number of ASSM as [1, 1, 2, 1, 1] to ensure effective feature extraction and low computational complexity. The sampling process is conducted by Rearrange operation, LN layer, and Linear layer, with the scale and channel of features undergoing a two-fold opposite change each time. Unlike the encoding process, we choose to up-sample the results of skip connections in the decoder. With the structure-explicit modeling $I_{C0}\in {\mathbb{R}}^{H\times W\times 1}$ and the region modeling $I_{R0}\in {\mathbb{R}}^{H\times W\times 1}$ extracted from ***I***, we construct a SGRF to guide the network in performing targeted enhancement of degraded textures and low-light regions. After two stages of feature reconstruction, the high-quality enhanced image $I_{E}\in {\mathbb{R}}^{H\times W\times 3}$ is refined by 1 × 1 convolution.

### 3.2 Adaptive state space module

During multi-dimensional mapping in deep networks, the degradation differences between feature channels are further amplified. However, due to the absence of an effective mechanism to capture interactions between channels, VMamba has limitations in correcting color deviations, which diminishes its practical effectiveness. Considering the above, we design the ASSM as shown in Figure 2 to assist in feature attention and region partitioning in the network. For the input features ***f***∈ℝ^*H*×*W*×*C*^, we first normalize them through a LayerNorm (LN) layer, and then use DS-CMM to capture channel dependencies. Unlike traditional channel attention, it adopts a dual-branch structure and captures channel interactions through different pooling mechanisms and multiple residual connections, which is computed as follows:


(1) $$\begin{array}{l} f_{CI}=f+f\times {\left(CLC\left(f_{A}\right)+CLC\left(f_{M}\right)\right)}_{Sig}, \end{array}$$

where ***f***_*A*_ and ***f***_*M*_ represent the features after average pooling and maximum pooling, respectively, and (·)_*Sig*_ is the sigmoid function. *CLC*(·) is a feature extraction module containing a LeakyReLU function placed between two convolutional layers. Next, the features ***f***_*CI*_ labeling the channel importance are fed into the vision state space module. The shallow features ***f***_*S*_ are obtained by LN and linear layers (Lin), while the deeper features $f_{S}^{\prime }$ are extracted by depth-wise convolution (DWC) and SiLU function:


(2) $$\begin{array}{l} f_{S}=\mathrm{Lin}\left(\mathrm{LN}\left(f_{CI}\right)\right),f_{S}^{\prime }={\left(\mathrm{DWC}\left(f_{S}\right)\right)}_{SiLU}. \end{array}$$

**Figure 3:**
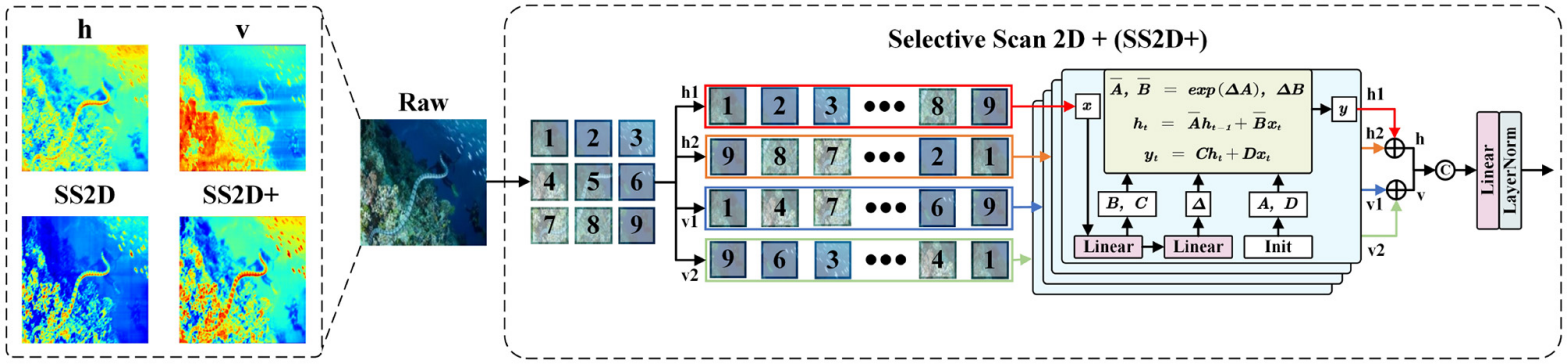
Specific flow of SS2D+. Unlike the original SS2D, we employ an improved strategy SS2D+ at the end, which discriminatively adds features from different directions and performs fusion learning. The heatmaps of horizontal adding (h) and vertical adding (v) results, SS2D, and SS2D+ are shown on the left.

Through the four-way scanning mechanism, VMamba overcomes the modeling limitations of SSM in 2D images and enhances the perception of non-causal features. The focus of this mechanism is to extend multiple scanning directions to capture dependencies. However, in practical underwater applications, we have observed notable directional properties in the modeling of horizontal and vertical directions. For instance, in the heatmaps of Figure 3, the horizontal scanning result highlights attention between horizontally adjacent texture features, while the vertical scanning result distinctly delineates the vertical division of image regions. Due to the differences between these two results, directly adding them not only interferes with the unique feature contributions, but also compromises the integrity of important feature modeling. To this end, we have introduced a softer fusion strategy SF to enhance the aggregation of features from different directions.

Following the settings in the four-way scanning mechanism, $f_{S}^{\prime }$ is serialized as a patch sequence and captures the interactions between sequence elements from four directions. To capture more accurate spatial remote dependencies, we employ an adding-fusion learning process SS2D+. Specifically, SS2D+ performs a discriminative addition on scanning results in the same direction, then superimposes the addition results from different directions at the channel level through concatenation. Further feature fusion is achieved through linear and LN layers, allowing the network to integrate the modeling properties of both directions, thereby highlighting the attention to important features:


(3) $$\begin{array}{l} f_{SS2D+}=LN\left(\mathrm{Lin}{\left(\left(f_{h1+h2}\right),\left(f_{v1+v2}\right)\right)}_{CAT}\right), \end{array}$$

where (***f***_*h*1+*h*2_) and (***f***_*v*1+*v*2_) represent the adding results in horizontal and vertical directions, respectively. Finally, the remote dependencies are labeled on the shallow feature ***f***_*S*_, and connected with ***f***_*CI*_ in a residual manner to obtain the final modeling result:


(4) $$\begin{array}{l} f_{ASSM}=f_{CI}+\mathrm{Lin}\left(f_{SS2D+}⊗\mathrm{Lin}{\left(f_{S}\right)}_{SiLU}\right). \end{array}$$

### 3.3 Structure-guided residual fusion module

**Figure 4:**
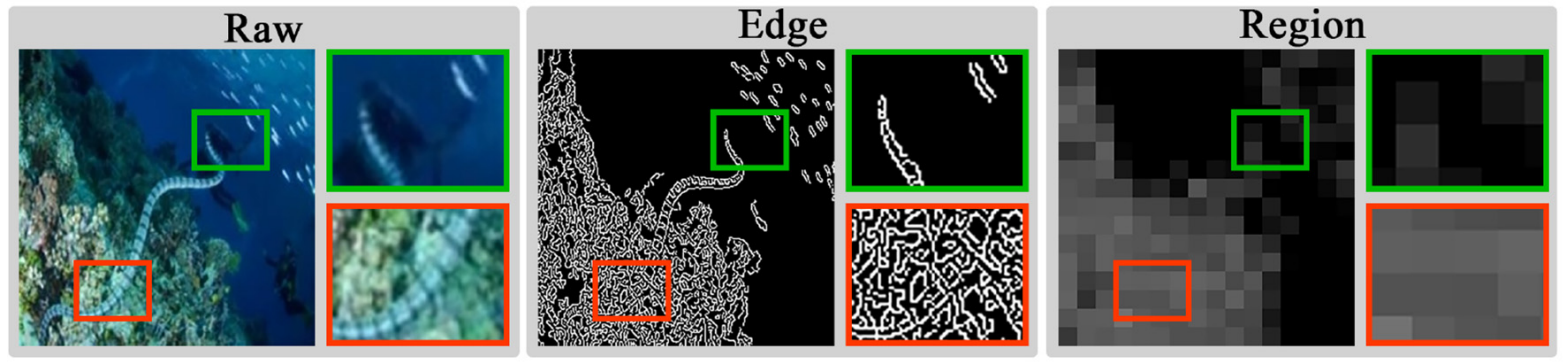
Modeling examples of different image regions. High-quality regions are framed in red and low-quality regions are framed in green.

As shown in Figure 4, explicit modeling of structural cues brings significant benefits in degraded scenarios, mainly reflected in two aspects: Firstly, edge modeling can directly highlight key textures in blurred details, which is highly effective in improving image clarity and contrast. Secondly, due to uneven brightness distribution, high-quality region modeling tends to yield richer feature information. By leveraging this property to distinguish regions with varying brightness, the model can more thoroughly understand the imaging structure and depth information. Due to the differences between encoded and decoded features, the fused features require a guiding mechanism to focus on degradation details and illumination loss. Based on structural cues, this mechanism can promote the model to enhance texture details and increase sensitivity to illumination changes, thereby further improving image enhancement performance.

**Figure 5:**
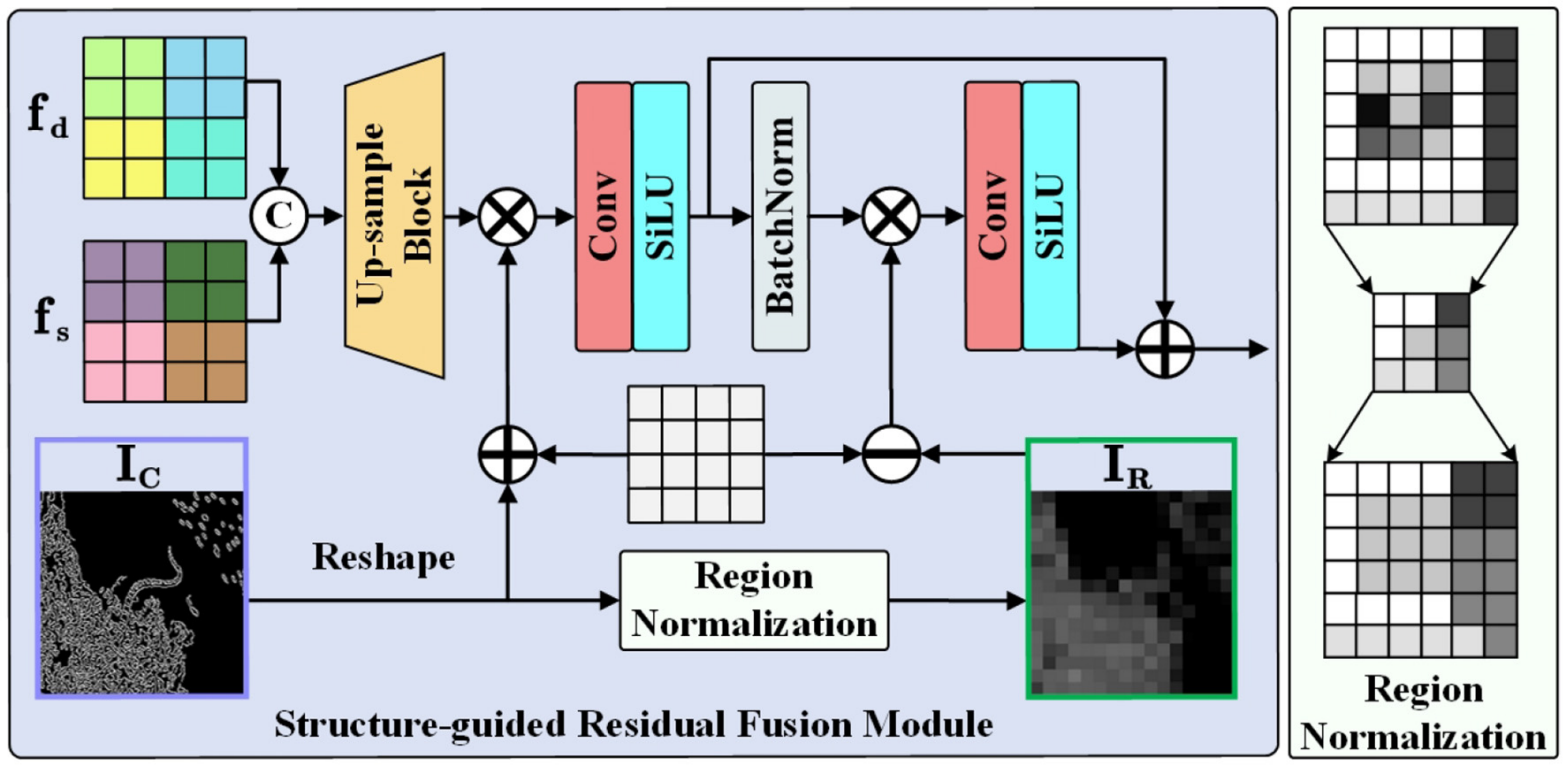
Structure of the structure-guided residual fusion module. With the region normalization shown on the right, we transform edge modeling ***I***_*C*_ into region modeling ***I***_*R*_ with rich feature distinctions.

To this end, we introduce a Structure-guided Residual Fusion Module (SGRF) during the decoding stage, the structure is shown in Figure 5. Firstly, SGRF generates an initial edge modeling $I_{C}\in {\mathbb{R}}^{H\times W\times 1}$ by the Canny operator (Liu et al., 2025) to guide the edge texture enhancement of the up-sampled fusion result. The process is represented as follows:


(5) $$\begin{array}{l} f_{\text{edge}}=CS\left(\left(\rho +I_{C}\right)*up{\left(f_{s},f_{d}\right)}_{CAT}\right), \end{array}$$

where ***f***_*s*_ and ***f***_*d*_ represent shallow encoded features and deep decoded features, and *up*(·) represents the up-sampling process. In addition, since black pixels will distort the original features, we set a constant matrix ***ρ*** to emphasize the edge information. All pixels of ***ρ*** are assigned a value of 1. After adding it to ***I***_*C*_, the original black pixel regions (i.e., pixels with a value of 0) exert no influence on the original features, while the texture distribution regions enhance the original features. Then, SGRF learns the edge enhancement feature ***f***_*edge*_ through convolutional layers and SiLU function (CS).

To fully utilize the structural modeling properties, we construct a clear illumination region division $I_{R}\in {\mathbb{R}}^{H\times W\times 1}$ by region normalization to guide the visibility improvement in low-light regions. The procedure first divides ***I***_*C*_ into multiple regions *r*_1_, *r*_2_, …, *r*_*i*_, *r*_*n*_ of size *R*×*R*, and then calculates the mean μ to replace the pixel value *p* of the region. The computation for the *i*-region is as follows:


(6) $$\begin{array}{l} \mu_{i}=\frac{1}{R^{2}}\underset{p_{ij}r_{i}}{\sum }p_{ij}. \end{array}$$

In region modeling ***I***_*R*_, regions with rich edge detail exhibit higher pixel values, which also implies low pixel values in low-light regions. We use the constant matrix ***ρ*** to invert the pixel distribution of the region. When ***ρ*** is subtracted from ***I***_*R*_, pixel values in low-light areas are amplified while those in bright regions are suppressed, thereby guiding the network to focus on regions with light illumination loss. The relevant process is as follows:


(7) $$\begin{array}{l} f_{SGRF}=f_{\text{edge}}+CS\left(\left(\rho -I_{R}\right)*BN\left(f_{\text{edge}}\right)\right), \end{array}$$

where *BN*(·) represents the BatchNorm layer. Finally, SGRF combines the two enhancement features by residual connection, enabling the network to enhance degraded details and low-light regions in a more targeted manner.

### 3.4 Loss functions

(1) Charbonnier Loss: As a variant of the *L*_1_ loss, the charbonnier loss varies more gently when the gradient is large, which helps to measure high-frequency details (e.g., edges, textures, etc.) between images. In addition, this loss is more robust to outliers, ensuring training stability.

(8) $$\begin{array}{l} L_{C}=\sqrt{||I_{E}-I_{GT}|{|}^{2}+\varepsilon^{2},} \end{array}$$

where ***I***_*E*_ and ***I***_*GT*_ represent enhanced and ground truth (GT) images, and ε represents a constant used to stabilize the loss, which is set to 10^−3^.
(2) Structural Similarity Index Measure (SSIM) Loss: Based on the measures of luminance, contrast and structure, the SSIM loss evaluates the image similarity from multiple perspectives, benefiting the network in generating visually enhanced images with improved brightness and structure.

(9) $$\begin{array}{l} L_{S}=1-\left(l\left(I_{E},I_{GT}\right)*c\left(I_{E},I_{GT}\right)*s\left(I_{E},I_{GT}\right)\right), \end{array}$$

where *l*(·, ·) is luminance similarity, *c*(·, ·) is contrast similarity, and *s*(·, ·) is structural similarity.

To train the proposed DWMamba, we adopt a combination of the two loss functions, aiming to encourage the network to generate enhanced results that are more similar to GT images in terms of detail and brightness. The total loss function is calculated as follows:


(10) $$\begin{array}{l} L_{\text{total}}=L_{C}+\lambda L_{S}, \end{array}$$

where λ represents the balance weight, which we set to 0.5 by default.

## 4 Experiments

### 4.1 Experiment setup

(1) Implementation details: We implemented DWMamba with the PyTorch 2.1.1 framework on a machine with an Intel Core i7-12700KF CPU, two NVIDIA GeForce RTX 4090 GPUs, and 64 GB of memory. The model was trained by the ADAM optimizer, with the learning rate set to 0.0001, the epochs to 100, and the batch size to 8.
During testing, DWMamba was compared with eleven advanced methods, including four traditional methods: TEBCF (Yuan et al., 2021), MLLE (Zhang et al., 2022), MSPE (Zhou et al., 2023b), and ICSP (Hou et al., 2024), six deep learning methods: TACL (Liu et al., 2022), PUGAN (Cong et al., 2023), TUDA (Wang et al., 2023), ASNet (Park and Eom, 2024), DDformer (Gao et al., 2024), and UVZ (Huang Z. et al., 2025), one mamba-based method: MambaIR (Guo et al., 2025). These methods were based on CNN, unsupervised techniques, Transformer, Mamba, and hybrid frameworks, respectively, and were experimented using the training models provided in the original paper. As MambaIR is not specifically designed for underwater image enhancement, we retrained it under same experimental configurations for fair comparison.
(2) Datasets and metrics: We randomly selected 810 pairs of real images from the UIEB (Li et al., 2019) dataset to train DWMamba, using the remaining 80 pairs as a reference test dataset. To verify the applicability, we also conducted extensive experiments on five non-reference datasets, namely OceanDark (Marques and Albu, 2020), UFO (Islam et al., 2020a), EUVP (Islam et al., 2020b), LNRUD (Ye et al., 2022), and NPE (Wang et al., 2013). The image sizes of all datasets were resized to 256 × 256 to facilitate the experiments.
For the referenced datasets, we used two full-reference metrics such as PSNR and SSIM to measure the similarity between the enhancement results and the GT images. Additionally, three non-reference metrics such as UCIQE (Yang and Sowmya, 2015), ENTROPY, and CEIQ (Fang et al., 2014) were used to evaluate the quality of all enhancement results.

### 4.2 Comparison on the reference dataset

**Figure 6:**
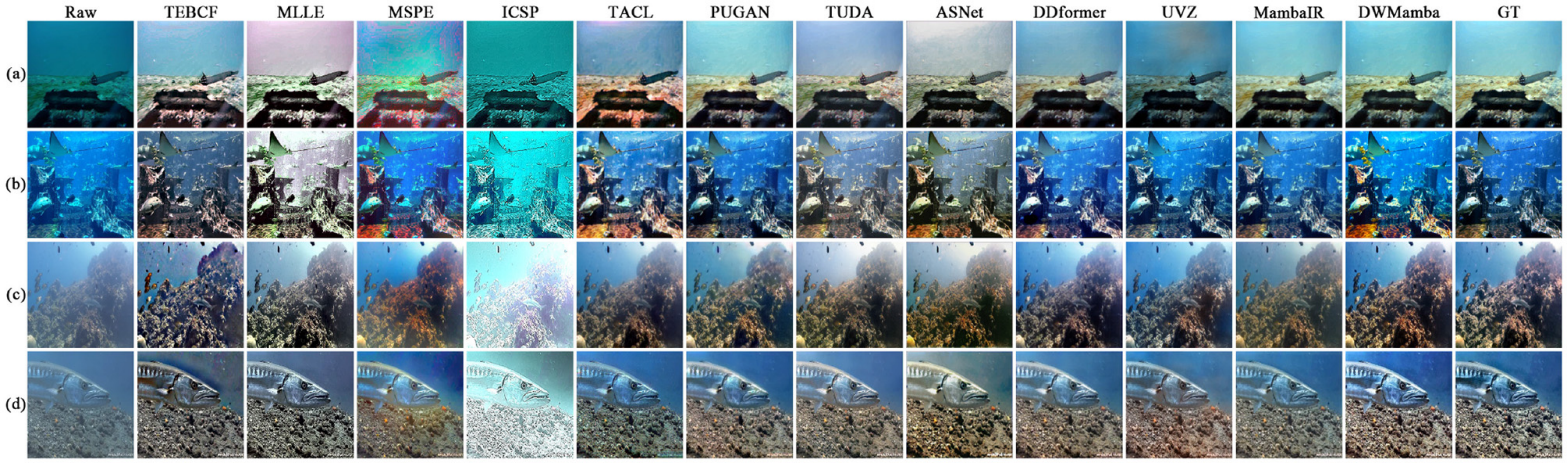
Comparison of enhancement results for all methods on the UIEB dataset. Each row represents a different scene, with variations in color and clarity reflecting distinct processing methods.

Figure 6 shows the enhancement examples of all methods on the UIEB dataset. The results from ICSP and ASNet exhibited varying degrees of overexposure, leading to oversaturated image colors. On the contrary, TEBCF and MLLE darkened the image brightness and masked the original image details, TEBCF and UVZ introduced artifacts in the enhancement process. The results from MSPE and TACL showed over-enhancement of the red channel, which disturbed the background color. The results of TUDA and MambaIR presented a relatively blurred scene, thereby reducing the color attractiveness of its results. PUGAN and DDformer effectively enhanced the image visibility, but the image details still need to be further enhanced. Our DWMamba demonstrated excellent performance in terms of color, clarity, and detail, particularly in the hazy scene (d), where the fish texture and scene colors were significantly enhanced.

**Figure 7:**
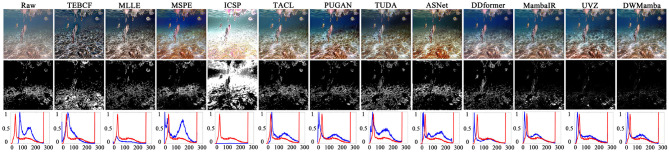
Comparison of residuals and red channel distribution between all enhanced images and GT. The first row shows the original images and the enhanced images from different methods, while the second row shows the corresponding residual images. The third row displays the red channel pixel distribution, with the blue and red lines representing the enhanced image and GT, respectively.

In Figure 7, we further compared the similarity between the different methods and GT using residual figures and red channel distribution figures. The white regions in the residual figures directly reflects the differences between the enhanced images and GT, while the distribution figures highlight the most damaged channels. Compared with the residual figures of the raw images, the white regions from TEBCF, ICSP, and ASNet were more extensive, indicating a more significant difference from GT. In contrast, UVZ and DWMamba had the smallest white regions, and the red channel distributions more closely matched those of GT, proving that the enhancement results were highly consistent with GT images.

Table 1 provides a performance metrics comparison for all methods on the UIEB dataset. Our method stands out among all methods for its competitive performance, lower complexity, and higher operational efficiency. Notably, DWMamba achieves optimal values in most of the metrics and is slightly behind TUDA and ASNet in Entropy, demonstrating its well-balanced trade-off between efficiency and effectiveness.

**Table 1:** Quantitative comparison for all methods on the UIEB dataset (optimal, sub-optimal).

	**TEBCF**	**MLLE**	**MSPE**	**ICSP**	**TACL**	**PUGAN**	**TUDA**	**ASNet**	**DDformer**	**UVZ**	**MambaIR**	**DWMamba**
PSNR↑	20.48	17.25	21.92	12.61	22.79	25.67	22.82	21.68	23.53	23.61	22.52	26.37
SSIM↑	0.883	0.739	0.890	0.585	0.825	0.895	0.927	0.876	0.889	0.935	0.786	0.953
UCIQE↑	0.630	0.616	0.630	0.603	0.629	0.629	0.594	0.608	0.614	0.621	0.608	0.633
ENTROPY↑	7.496	7.547	7.405	6.945	7.557	7.572	7.594	7.599	7.491	7.511	7.425	7.591
CEIQ↑	3.433	3.469	3.369	3.159	3.457	3.474	3.479	3.482	3.412	3.441	3.384	3.488
Params (M)	-	-	-	-	11.37	95.65	31.36	0.755	7.580	5.387	0.626	0.430
FLOPs (G)	-	-	-	-	56.86	72.05	174.4	48.97	17.87	499.5	10.21	5.018
Times (s)	1.479	0.072	0.176	0.083	0.658	0.438	0.051	0.030	0.033	0.122	0.037	0.028

### 4.3 Comparison on the non-reference datasets

**Figure 8:**
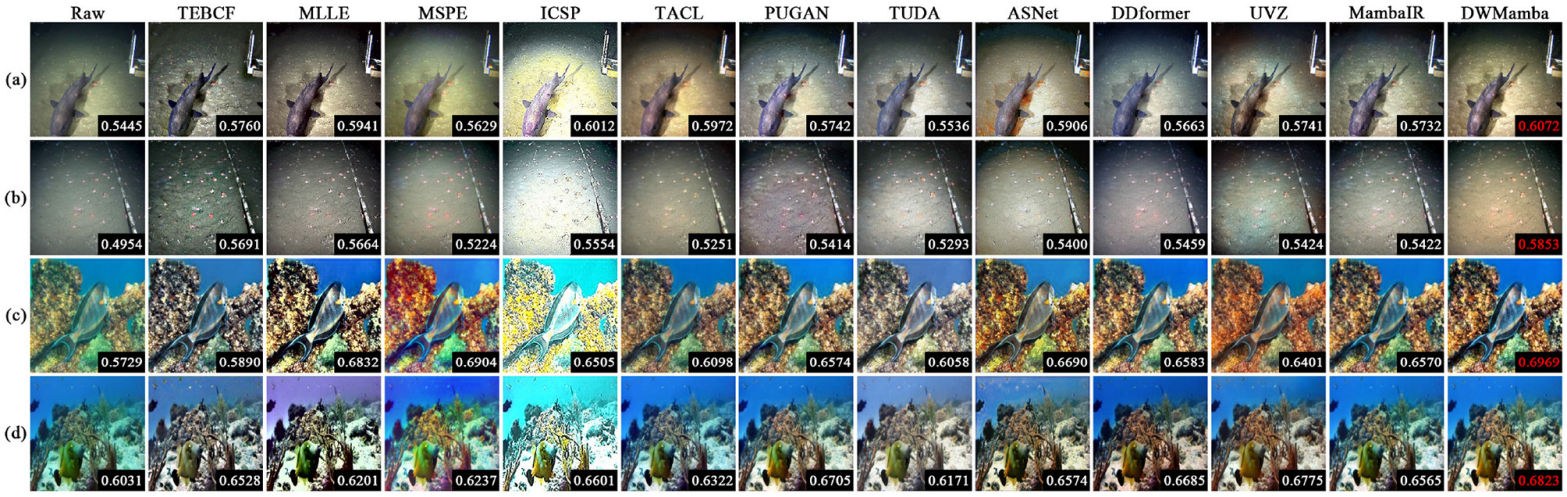
Comparison of enhancement results and UCIQE for all methods on the OceanDark and UFO datasets, with red values indicating optimal metrics. Each row (AD) depicts a different underwater scene.

In this section, we present the performance of all methods on the four non-reference datasets. Figure 8 visualizes the enhancement effects and UCIQE metrics of the different methods on the OceanDark and UFO datasets. For dark scenes in OceanDark, most methods overexposed or darkened the brightness and failed to reasonably enhance the scene visibility. In contrast, TACL, ASNet, and our DWMamba effectively enhanced the brightness and contrast of the scene. However, TACL resulted in textures blurred by hazy lighting, while ASNet excessively enhanced the red channel in shadow regions. The UFO dataset highlights the color deviation and hierarchical scenes. TEBCF, MLLE, and ASNet rendered distant scenes less colorful, with even MLLE producing an illogical purple background. MSPE, UVZ, and MambaIR significantly enhanced color saturation, but there were still noticeable color deviations. PUGAN and DWMamba not only effectively enhanced color attractiveness while retaining well-defined scene boundaries, and DWMamba achieved the best UCIQE with the most vibrant colors.

**Figure 9:**
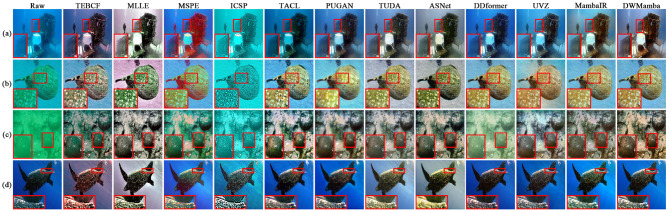
Detail enlargement comparison for all methods on the EUVP and LNRUD Datasets. Rows labeled (AD) show images of different underwater objects.

Figure 9 enlarges the enhanced details for the EUVP and LNRUD datasets. ASNet introduced unnatural yellow colors, significantly deviating from the true color distribution. The results of ICSP generally suffered from over-rendering of cyan, while MSPE and TEBCF excessively enhanced the red channel, and these over-saturated colors severely interfered with textural appearance. On the other hand, MLLE reduced the overall brightness of the image, making the enlarged details less clear. Although TACL and PUGAN considerably improved the visual effect of the underwater scene, the enlarged details were still affected by color deviation. The enhanced results of MambaIR demonstrated excellent color reproduction, but introduced other colors and blurred details. Compared to the above methods, TUDA and DWMamba significantly improved scene visibility, with the enlarged texture presenting clearer details. As shown in Table 2, our DWMamba achieved both optimal and sub-optimal values on the four unreferenced datasets, further proving its excellent enhancement effect and wide applicability. In addition, TUDA achieves outstanding performance in ENTROPY and CEIQ through excellent brightness enhancement and detail restoration.

**Table 2:** Quantitative comparison for all methods on the four non-reference datasets (optimal, sub-optimal).

**Dataset**	**Metric**	**TEBCF**	**MLLE**	**MSPE**	**ICSP**	**TACL**	**PUGAN**	**TUDA**	**ASNet**	**DDformer**	**UVZ**	**MambaIR**	**DWMamba**
OceanDark	UCIQE↑	0.584	0.583	0.568	0.596	0.591	0.580	0.556	0.573	0.572	0.588	0.574	0.598
	ENTROPY↑	7.377	7.456	7.621	6.929	7.709	7.559	7.581	7.528	7.570	7.619	7.683	7.735
	CEIQ↑	3.354	3.369	3.526	3.183	3.556	3.459	3.473	3.455	3.437	3.489	3.557	3.577
UFO	UCIQE↑	0.628	0.626	0.632	0.627	0.624	0.627	0.591	0.617	0.617	0.610	0.622	0.640
	ENTROPY↑	7.450	7.533	7.438	7.037	7.453	7.509	7.584	7.510	7.394	7.288	7.462	7.541
	CEIQ↑	3.397	3.425	3.375	3.252	3.378	3.420	3.465	3.424	3.337	3.279	3.398	3.445
EUVP	UCIQE↑	0.616	0.617	0.621	0.631	0.621	0.614	0.585	0.593	0.601	0.612	0.605	0.621
	ENTROPY↑	7.619	7.329	7.465	6.679	7.646	7.615	7.654	7.588	7.533	7.554	7.539	7.662
	CEIQ↑	3.502	3.330	3.401	3.159	3.510	3.489	3.516	3.474	3.428	3.449	3.450	3.530
LNRUD	UCIQE↑	0.626	0.615	0.628	0.610	0.624	0.625	0.593	0.613	0.612	0.608	0.618	0.634
	ENTROPY↑	7.504	7.552	7.463	7.102	7.533	7.551	7.611	7.557	7.454	7.393	7.489	7.580
	CEIQ↑	3.432	3.447	3.393	3.289	3.433	3.451	3.484	3.457	3.380	3.347	3.417	3.472
NPE	ENTROPY↑	7.286	6.706	7.399	6.172	7.265	7.461	7.309	7.247	7.152	7.348	7.542	7.546
	CEIQ↑	3.279	2.904	3.374	3.068	3.256	3.376	3.309	3.263	3.165	3.314	3.475	3.476

**Figure 10:**
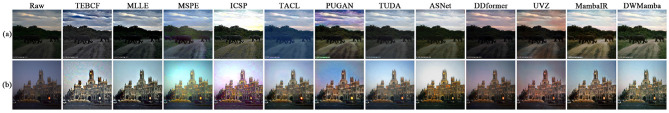
Comparison of enhancement results for all methods on the NPE dataset. Row (A, B) display different low-light outdoor scenes.

In addition, Figure 10 presents the enhancement results of all methods on the low-light NPE dataset. It can be observed that most methods fail to effectively improve the overall brightness, resulting in unclear and dim outputs. The results of MSPE and ICSP suffer from oversaturation and overexposure, which obscure fine details. PUGAN, DDformer, and UVZ excessively amplify the red channel, leading to noticeable color distortions. In contrast, MambaIR and DWMamba consistently enhances both brightness and contrast in low-light scenes while preserving natural color tones without introducing artifacts. For quantitative evaluation, since UCIQE is specifically designed for underwater scenarios, only ENTROPY and CEIQ were adopted for the NPE dataset. The results demonstrate that DWMamba achieves the best performance on both metrics.

### 4.4 Ablation study

**Figure 11:**
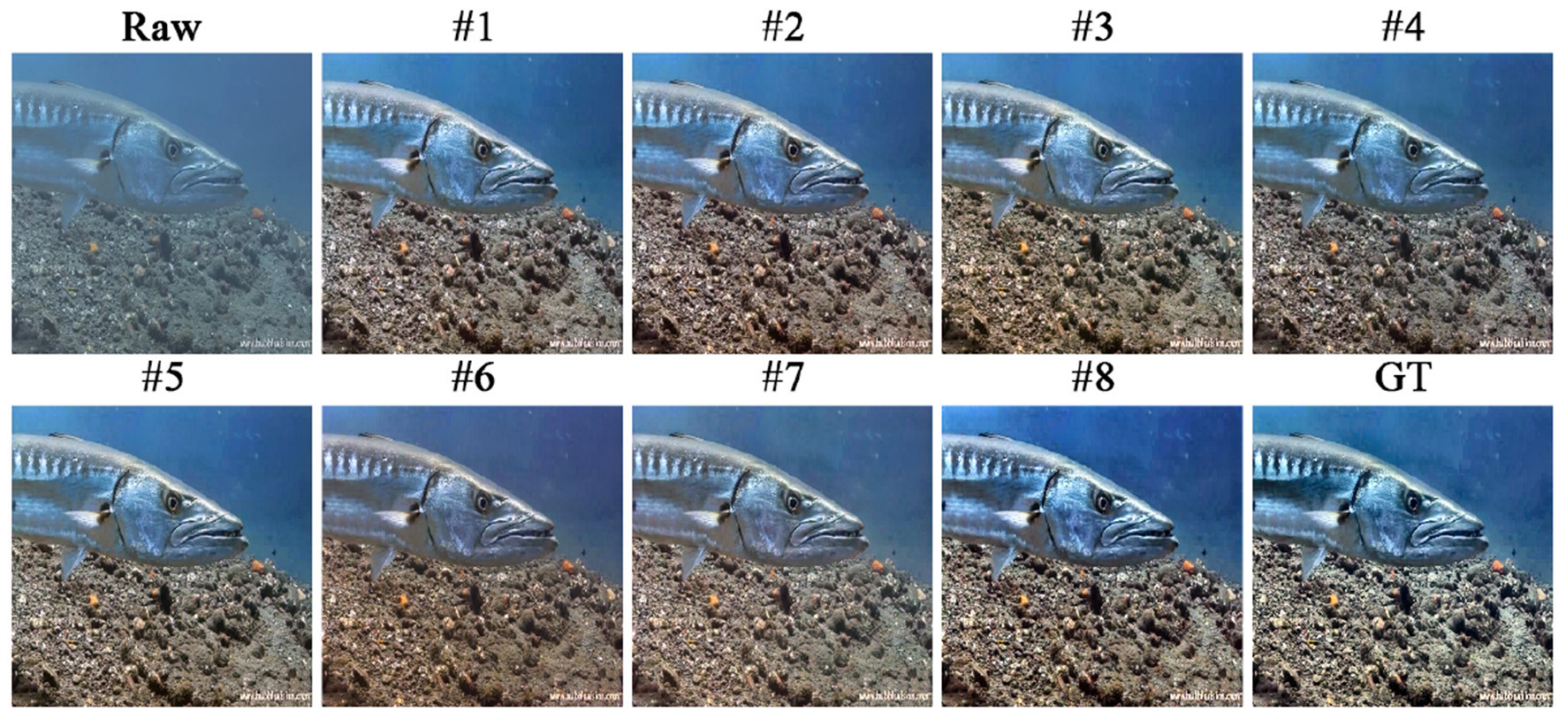
Enhanced results of all ablation configurations.

In Table 3, we provided a detailed configuration and analysis of the key components of DWMamba, using the UNet architecture and the original VMamba as a baseline (#1). Figure 11 illustrates that the network with complete components produces more prominent texture details and more natural background color, effectively avoiding color deviation and the introduction of other colors. In contrast, in the absence of SF, i.e. using the original SS2D, that is, when using the original SS2D, the background exhibits excessive enhancement of the red channel, while the overall brightness of the image decreases. Without DS-CMM or SGRF, the fish texture and the background exhibit an uncorrected blue deviation, highlighting deficiencies in region division. When lacking both DS-CMM and SF, i.e. using the original VSSM, the enhanced results showed blurred textures and dull colors. This highlights the importance of more comprehensive feature dependency modeling. As shown in Table 3, the complete network achieves the most outstanding metric performance, indicating that all three components are beneficial for the performance improvement of the baseline model. Notably, the absence of SGRF results in the most significant performance degradation, demonstrating its effectiveness in focusing on degradation details and illumination loss.

**Table 3:** Component settings and metrics comparison for all ablation configurations (optimal, sub-optimal).

**Configuration**	**#1**	**#2**	**#3**	**#4**	**#5**	**#6**	**#7**	**#8**
Base	✓	✓	✓	✓	✓	✓	✓	✓
DS-CMM		✓			✓	✓		✓
SF			✓		✓		✓	✓
SGRF				✓		✓	✓	✓
PSNR↑	24.88	25.70	25.90	26.17	26.22	26.36	26.11	26.37
SSIM↑	0.941	0.945	0.946	0.948	0.943	0.949	0.949	0.953
UCIQE↑	0.621	0.624	0.625	0.621	0.622	0.627	0.628	0.633
ENTROPY↑	7.571	7.547	7.570	7.535	7.545	7.556	7.585	7.591
CEIQ↑	3.479	3.465	3.475	3.454	3.456	3.468	3.486	3.488

**Figure 12:**
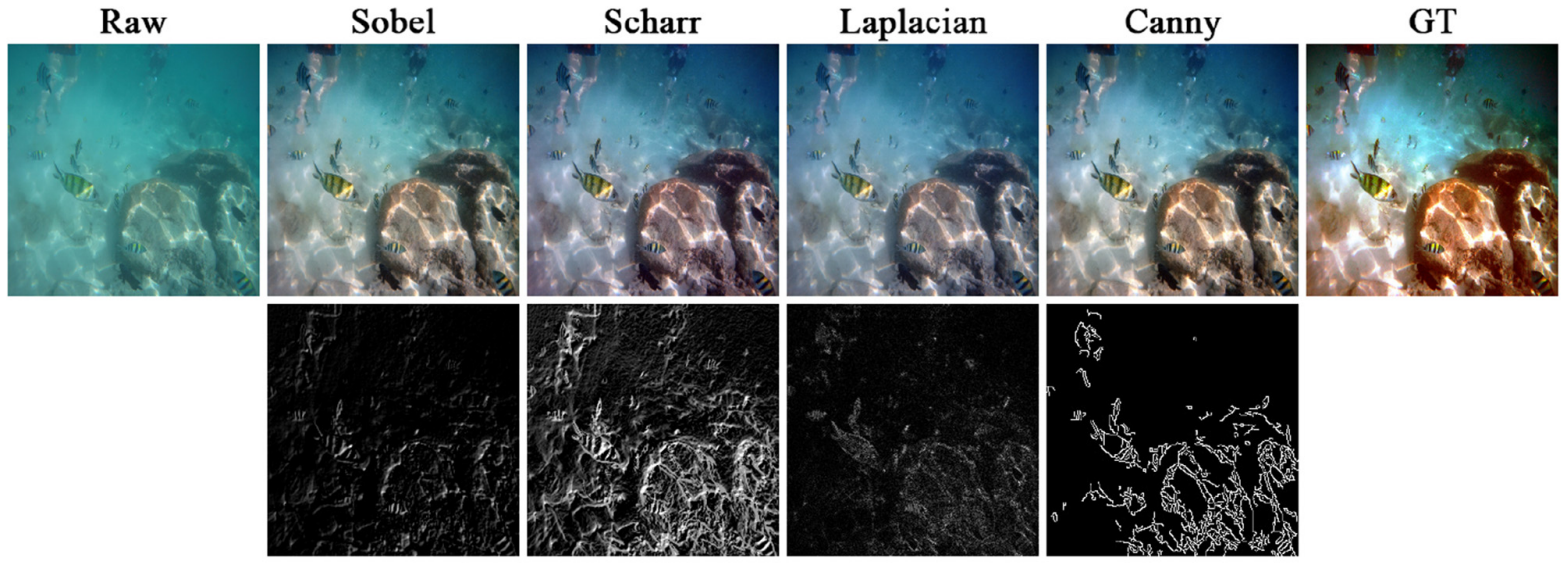
Comparison of results using different operators. The top shows the raw image and the enhanced results using different operators, and the bottom shows the corresponding edge detection results.

To verify the impact of different operators (Su et al., 2021) on the network performance, we adopted Sobel, Scharr, Laplacian, and Canny operators to construct the edge modeling of the raw image, and employed region normalization for subsequent region modeling. Figure 12 and Table 4 compare the enhancement results and metrics using different operators, respectively. Specifically, the network with the Sobel operator effectively improves the visual quality, but fails to completely eliminate the color deviation. The network using Scharr and Laplacian operators generated hazier enhancement results, accompanied by significant red over-enhancement. The network using the Canny operator generates superior results in terms of color and clarity, achieving optimal or sub-optimal evaluations across all metrics. Further analyzing the edge detection results, we found that the edge information from Sobel and Laplacian operators were relatively weak and cannot clearly reflect the rich details of the actual scene. Scharr and Canny operators generated more prominent edge information, but Scharr introduced substantial scene noise, which was detrimental to subsequent region division. Therefore, we prioritized the Canny operator for edge modeling.

**Table 4:** Metrics comparison of networks using different operators (optimal, sub-optimal).

	**PSNR↑**	**SSIM↑**	**UCIQE↑**	**ENTROPY↑**	**CEIQ↑**
Sobel	25.92	0.948	0.630	7.609	3.501
Scharr	26.29	0.950	0.628	7.583	3.482
Laplacian	26.27	0.949	0.627	7.532	3.450
Canny	26.37	0.968	0.633	7.591	3.488

**Figure 13:**
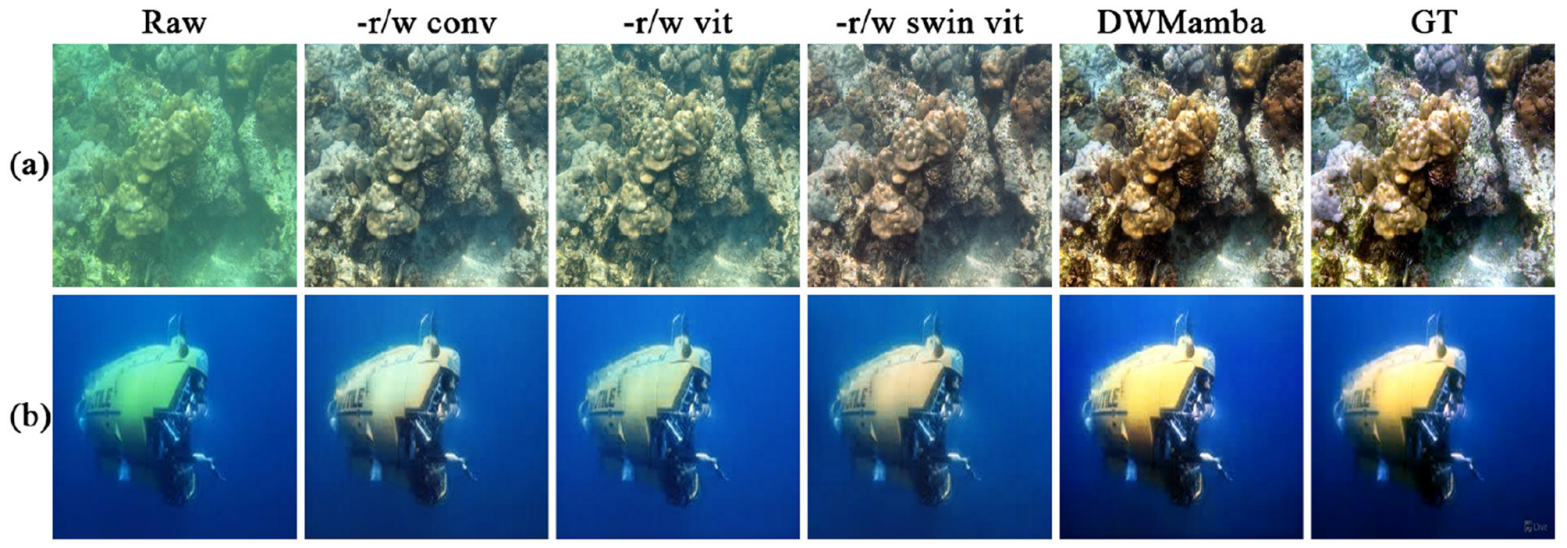
Comparison of results using baseline model. Row (A, B) display classic blue and green deviation scenes.

Focusing on baseline model and model size, we conducted an ablation study as shown in Table 5. First, we fixed the remaining module configurations and replaced the core module ASSM with a single convolutional layer (-r/w conv), Vision Transformer (-r/w vit), and Swin Transformer (-r/w swin vit), where -r/w conv can also be regarded as the network without ASSM. Furthermore, we design small-scale configurations S: (layers: [1, 1, 2, 1, 1], dimensions: [24, 48, 96, 48, 24]) and large-scale configurations L: (layers. [2, 2, 9, 2, 2], dimensions: [48, 96, 192, 96, 48]). Among these, DWMamba-S is the main experimental architecture in this paper. Based on the relatively good performance of -r/w swin vit, DWMamba-S achieved a significant reduction of 41.8% in parameters and 36.2% in FLOPs, and increased PSNR by 16.7%. Similarly, DWMamba-L reduced parameters by 40.1%, FLOPs by 39.1%, and increased PSNR by 7.2%. The results in Figure 13 further demonstrate the visual advantages of DWMamba over other baseline models.

**Table 5:** Metrics comparison of different baseline model and model size (optimal, sub-optimal).

	**PSNR↑**	**SSIM↑**	**UCIQE↑**	**ENTROPY↑**	**CEIQ↑**	**Parameter (M)**	**FLOPs (G)**
-r/w conv-S	22.22	0.916	0.583	7.358	3.330	0.324	4.400
-r/w vit-S	21.69	0.904	0.575	7.289	3.281	0.627	7.386
-r/w swin vit-S	22.59	0.921	0.592	7.352	3.337	0.694	7.876
DWMamba-S	26.37	0.953	0.633	7.591	3.488	0.404	5.018
-r/w conv-L	21.86	0.919	0.614	7.543	3.451	3.826	32.41
-r/w vit-L	22.30	0.910	0.581	7.338	3.312	8.139	62.45
-r/w swin vit-L	24.98	0.944	0.619	7.523	3.445	9.547	71.25
DWMamba-L	26.80	0.953	0.620	7.549	3.465	5.715	43.32

nOTABLY, although DWMamba exhibits significant advantages in all metrics, the marginal gains in performance diminish when scaled to large-scale model configurations, especially in the non-reference quality assessment metrics. In this regard, we believe that although more complex model architectures enhance the model's ability to approximate GT images, the information loss of deeper architectures and the inherent limitations of GT data constrain the performance improvement. Therefore, in future research, striking a balance between model complexity and feature extraction capability will be the essential direction to further improve the performance of DWMamba and its similar models.

To assess the computational complexity of DWMamba, we compared the model's FLOPs and inference time across image inputs of varying sizes. As shown in Table 6, when the width and height of the input image doubled, the FLOPs increased nearly fourfold, aligning with the linear distribution of pixel count growth. This conclusively demonstrates that DWMamba inherits the linear complexity characteristic of the State Space Model, without introducing quadratic overhead.

**Table 6:** Comparison of computational complexity at different image sizes.

**Size**	**64**	**128**	**256**	**512**	**1024**
FLOPs (G)	0.3136	1.2546	5.0182	20.073	80.2916
Time (s)	0.3830	0.4115	0.4116	0.5110	1.1534

### 4.5 Generalizability test

**Figure 14:**
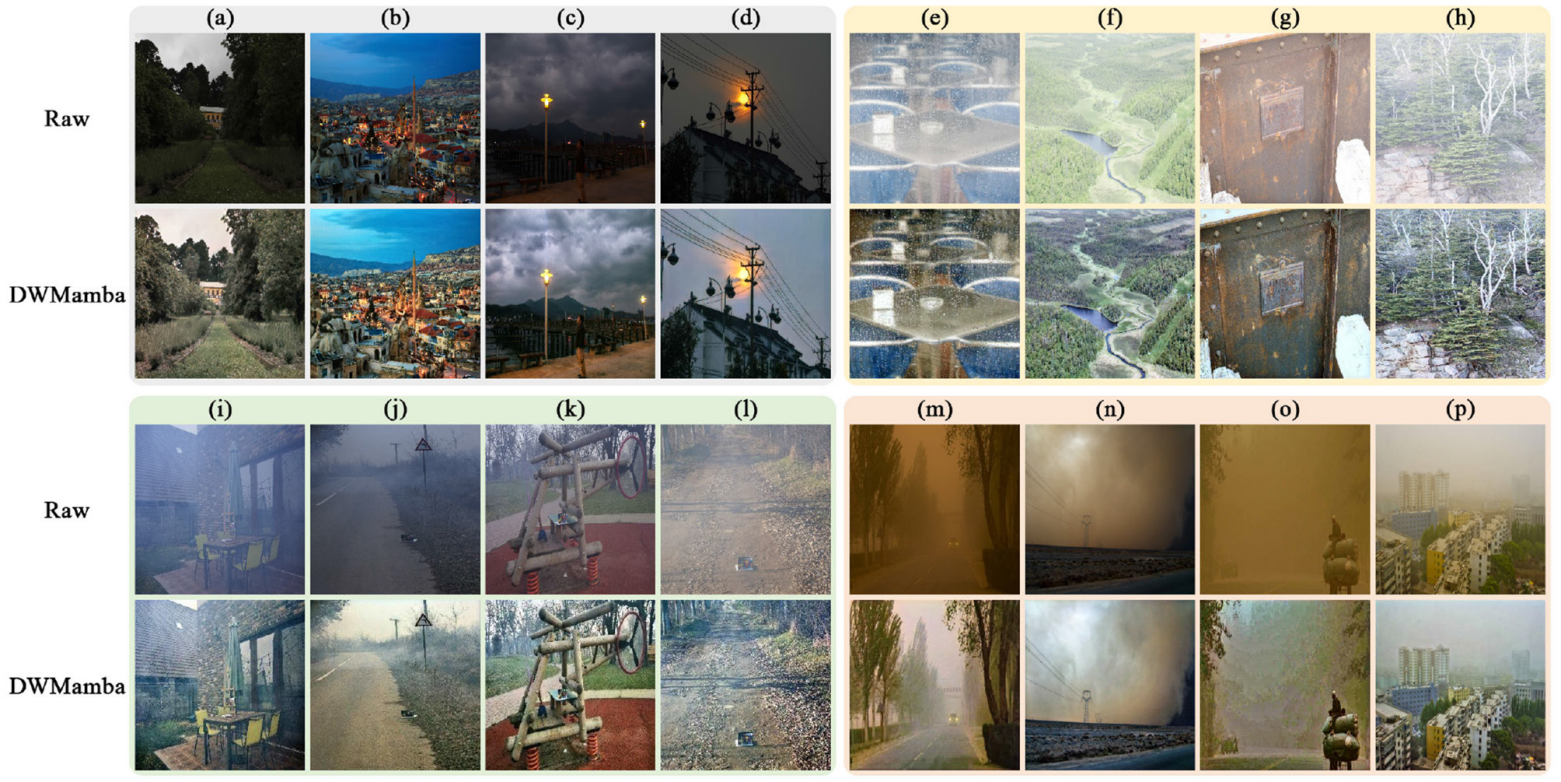
Generalizability of DWMamba across diverse poor-lighting scenarios. **(a–d)** Are low-light images from the NPE (Wang et al., 2013) dataset, **(e–h)** are overexposure images from the MIT (Bychkovsky et al., 2011) dataset, **(i–l)** are haze images from the O-HAZE (Ancuti et al., 2018) dataset, and **(m–p)** are sandy images from the WEAPD (Xiao et al., 2021) dataset. The results clearly demonstrate that DWMamba possesses domain-agnostic properties.

The complex and variable lighting conditions pose a challenge to IQI algorithm's generalization performance. Specifically, this challenge is reflected in multiple aspects: insufficient brightness due to light attenuation, overexposure from artificial light sources, image hazing due to transmission media, and visual interference from suspended particles, all of which significantly impact image quality. To this end, we conducted generalizability tests on four corresponding land datasets, and applied the DWMamba model without parameter tuning to these scenarios. As shown in Figure 14, for the low-light and overexposed scenarios, DWMamba demonstrates excellent adaptive brightness adjustment, making the dull colors and textures more attractive. For the hazy and sandy scenarios with more complex degradation, DWMamba does not completely eliminate the adverse effects of these extreme environments, but still shows significant visual improvement and plays a positive role in enhancing image visibility and scene understanding.

### 4.6 Limitation

While DWMamba achieves strong results across various real-world scenarios, it struggles with severe degradation. When images lose most of their color and detail, the enhancement task shifts toward generation, and our method fails to produce satisfactory visibility. Future research will focus on expanding multi-source information fusion for underwater scenes and advancing its real-world application.

## 5 Conclusion

In this paper, we present DWMamba, a novel adaptive Mamba network for image quality improvement. Specifically, we introduce a dual-stream channel monitoring mechanism and a soft fusion mechanism in the vision state space model, significantly enhancing its ability to capture global dependencies. In addition, we establish explicit structural and regional modeling to facilitate the targeted fusion of shallow and deep features. Extensive experiments across various datasets demonstrate that DWMamba exhibits not only excellent generalization but also significant quality improvements under diverse extreme lighting conditions. Notably, this multi-scenario applicability comes without high computational costs, highlighting its potential for practical applications.

## Data Availability

The raw data supporting the conclusions of this article will be made available by the authors, without undue reservation.
